# Implementation and evaluation of whole-course-based Internet Hospital Outpatient Pharmacy Services: a cross-sectional study in western China

**DOI:** 10.3389/fpubh.2024.1448471

**Published:** 2024-12-13

**Authors:** Yangyang Gao, You Lv, Shiyan Wang, Mengran Guo, Yi Guo, Minglin Zheng, Lulu He, Fengbo Wu, Ping Fan

**Affiliations:** ^1^Department of Pharmacy, West China Hospital, Sichuan University, Chengdu, China; ^2^Information Technology Center, West China Hospital, Sichuan University, Chengdu, China

**Keywords:** Internet Hospital, Internet Hospital Outpatient Pharmacy Services (IHOPSs), internet plus healthcare, COVID-19, pharmaceutical service

## Abstract

**Background:**

In recent years, the development of telemedicine and eHealth services has led to the rapid worldwide growth of Internet hospitals, which played a significant role during the coronavirus disease 2019 (COVID-19) pandemic. However, little is known about the characteristics and safety of Internet hospital outpatient pharmacy services (IHOPSs), which represent a new model of pharmaceutical services.

**Objective:**

This study aimed to reveal the comprehensive characteristics and safety of whole-course-based IHOPSs in a general tertiary hospital in western China.

**Methods:**

We established a whole-course–based IHOPS model. A total of 373,936 online prescriptions placed from February 1, 2020 to January 31, 2023 were analyzed. These included information on patients, prescriptions, and deliveries; error rates for prescription reviews and medication dispensations; economic value; and degree of patient satisfaction. Over the course of the study, a total of 373,936 prescriptions representing 351,884 patients and 945,172 medications were delivered to 22 provinces, 5 autonomous regions, and 4 municipalities in China.

**Results:**

IHOPSs saved patients more than 320,376 days (7,689,036 h) and RMB (Renminbi) ¥94.05 million in costs. The error rates of prescription review and dispensing were 0.0011% and 0.0008%, respectively. The infectious disease department (*n* = 63,903; 17.09%) ranked first in the number of prescriptions written for all three consecutive years. Of the 373,936 delivered prescriptions, 90.15% (337,104/373,936) were sent to Sichuan.

**Conclusion:**

The IHOPS was found to be efficient, convenient, and safe because it handled the challenge of precisely and safely delivering medications to patients on time during and to the end of the COVID-19 pandemic. It provided patients with safe and convenient pharmaceutical services unlimited by geography or time zones. Widespread use of this service could help alleviate pressure on offline pharmacists, giving them the time and resources to provide other professional services. Our model can therefore serve as a useful reference for policymakers to support the development of Internet pharmaceutical services. Further efforts are needed to regulate and standardize the management of this novel service.

## Introduction

Telemedicine and eHealth services are rapidly expanding technologies that promote quality in healthcare worldwide (1). However, China faces the challenge of limited medical resources, with high-quality services being concentrated in large cities. To solve these problems, the Chinese government has begun to promote Internet health services as a novel model of healthcare, enacting various relevant policy interventions to promote integration of the Internet with healthcare and aiming to increase access to and improve healthcare quality and efficiency (2). For instance, the “Opinion on Promoting the Development of Internet + Medical and Health Care” issued by the State Council of the People's Republic of China (PRC) allows medical institutions and drug distributors to entrust qualified third-party institutions that have been approved by pharmacists to distribute prescription treatments for chronic diseases online.

Outpatient pharmacy service is one part of the hospital health care system and it delivers drug-related health care for outpatients. Patients who are satisfied with outpatient pharmaceutical services are more likely to take medications properly and have drug adherence. So, delivering quality outpatient pharmaceutical service can enhance the satisfaction of patients and obtain the expected treatment outcome (3). Outpatient pharmacy service is crucial for safe and high-quality treatment. Pharmacists, knowledgeable and skilled professionals in medication management, disease prevention, and drug-related problems, can offer vital interventions that contribute to improved patient outcomes (4). Additionally, pharmacist-led interventions, especially medication rationality, have been shown to reduce the incidence of medication errors (4). Advances in healthcare and information technology have expanded pharmacists' professional roles and made them essential in healthcare (4).

The coronavirus disease 2019 (COVID-19) pandemic spawned explosive growth of Internet hospitals (5). During the pandemic, the strict quarantine policy deterred most patients from seeking offline healthcare (6). For those with chronic diseases requiring long-term medication use, at-home quarantine without consecutive medication can lead to disease exacerbation. Internet hospital pharmaceutical services improve the accessibility of quality pharmaceutical resources, helping to re-distribute these resources and transform the structure of the pharmaceutical system. COVID-19 significantly changed how people fill prescriptions: during the pandemic, Internet hospitals attracted many patients due to their convenience and accessibility. Previously, the research mainly focused on the traditional model of outpatient pharmaceutical care. However, few studies have been conducted on how safely Internet hospitals prescribe medications. As COVID-19 pandemic controls have loosened (7), establishment of a model assessing the quality and safety of Internet hospital pharmaceutical care as a long-term medical service is urgently needed.

In February 2020, the West China Hospital of Sichuan University (WCHSU; Chengdu, China) officially opened an Internet hospital. Using intelligent pharmaceutical thinking, information technology, and logistics, they created a model called “Internet Hospital Outpatient Pharmacy Services” (IHOPSs), which focuses on the safety of drug dispensing and delivery. In this model, doctors prescribe medications online, and pharmacists handle the process and logistics of dispensing and distribution.

This 3-year study aimed to further determine the effectiveness of using pharmaceutical services in IHOPSs and establish a high-quality, safe, and standardized model of operation for hospitals. We collected data related to Internet medicine at WCHSU from February 2020 through February 2023 to discover the characteristics, economic value, medication-dispensing errors (MDEs), and prescription review errors of the IHOPSs. We also aimed to assess whether using the IHOPSs could reduce the burden on the healthcare system and how medication safety for IHOPS patients could be ensured.

## Materials and methods

### Data collection

The descriptive cross-sectional study design was conducted. We obtained detailed information on online prescriptions from the hospital information system (HIS) of WCHSU from February 2020 through February 2023. As of February 2023, the system has processed >370,000 Internet prescriptions, providing pharmacy services to >350,000 people across China. The principal investigator (O.M.I.) were trained to ensure the validity and practicality of the research methods, and they were supervised during data collection. Patients' gender, age, associated prescription departments, payment, time of prescription, and drug delivery region were included in the analysis.

### Design of the whole-course-based Internet hospital pharmacy service model

**Figure 1:**
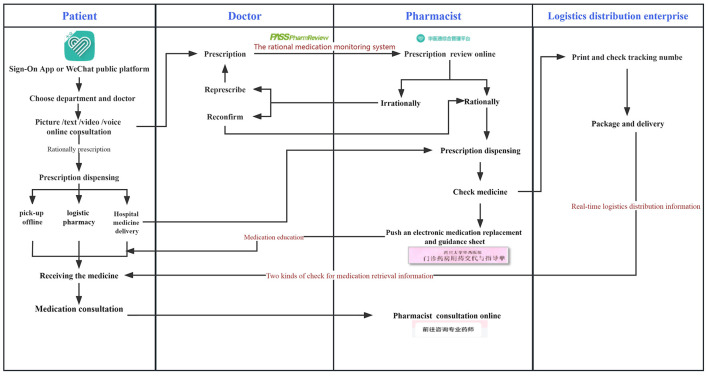
Flowchart of the IHOPS process.

The IHOPSs investigated in this study were available 24 h a day. Figure 1 shows the flowchart of the IHOPS process. Patients with long-term prescriptions for chronic diseases could obtain online consultations at the Internet hospital using our WeChat public platform or app via a mobile phone, and doctors could issue prescriptions online.

Pharmacists used a rational medication monitoring system developed by Sichuan Medicom Software Co. Ltd. (Chengdu, China) to review prescriptions in accordance with relevant laws and regulations. The prescription review process is shown in Figure 1. To ensure the accuracy of prescriptions, this process included three steps: rational medication monitoring system review, a pharmacist review with an certification authority (CA) signature, and a double check when dispensing. Suspicious or otherwise inappropriate prescriptions were queried or rejected and then recorded. All prescriptions were required to meet 100% of qualifications before they go live.

After prescription review was completed, the patient paid the fee and chose how to obtain their medication: home delivery, pickup at a hospital outpatient pharmacy, distribution to a social retail pharmacy, or purchasing with an electronic prescription. Most patients opted for delivery to the hospital pharmacy. Pharmacists checked the medication, patient, and logistics information and then arranged delivery by the entrusted logistics company. Patients could receive medications if they were able to pass more than two identification check methods. If a medication needed to be stored at 2–8°C, logistics personnel needed to record the in transit-temperature record for the drug, and the patient signed for the drug after confirmation of the temperature.

After receiving their medication, the patient received an electronic medication guide from the pharmacist to help them properly take their medicine. If they had any problems with the medication, they could consult a pharmacist online. The IHOPS thus provided patients with start-to-finish pharmaceutical care.

### Relevant qualifications and range of medications

In accordance with national prescription-related laws and regulations, the pharmaceutical professional and technical personnel who dispense prescriptions needed to undergo qualification review and training.

The entrusted logistics distribution enterprise conducted a qualification review and record, obtained relevant qualification documents according to the “Drug Management Quality Management Standard,” and signed the entrusted-transportation agreement as needed.

Sales of medications through the Internet complied with the Drug Administration Law of the People's Republic of China, the Administrative Measures for Internet Hospitals (Trial Implementation), and other relevant laws and regulations. The Internet hospital affiliated with our hospital does not sell some varieties of medications, including vaccines, blood products, narcotic drugs, psychotropic drugs, toxic drugs for medical use, radioactive drugs, and drug precursor chemicals. Current Internet hospital sales of medications accounted for 66.25% (693/1,046) of all offline products.

### Establishment of a medication quality and safety system

To ensure the safety and effectiveness of patient medications, we explored an established drug quality and safety system.

Every prescription was double-checked; if the dispensing pharmacist made an error, the reviewing pharmacist could intercept it. Meanwhile, the reviewing pharmacist records the dispensing pharmacist's errors, which are included in such documentation as performance reviews and statistical analyses. These actions form the basis of an internal dispensing error prevention and control system.

We check dispensing errors daily by taking inventory of medications. Once an inconsistency is found, immediately call the camera to view, implement the error, and correct it on time. Furthermore, we strengthen medication management by means of self-education and own-error analysis and assessment, provide case analysis and training for all employees, and provide detailed performance appraisals.

### Data calculation

We coded disease diagnoses in accordance with the International Classification of Diseases, version 10 (ICD-10). Drug delivery distance is calculated from the hospital to the mailing destination.

Following the method used by Yang et al. (8), we converted time costs into lost wages, calculating the cost of transport to and from the hospital based on second-class high-speed railway fare for the patient. The formula for economic cost savings per patient is as follows:


$$\begin{array}{l} \text{wage loss}+2\times \text{train ticket fee}. \end{array}$$

### Statistical analysis

We used Microsoft Excel 2020 (Microsoft Corp., Redmond, WA, USA) and SPSS software version 21.0 (IBM Corp., Armonk, NY, USA) for data storage and analysis. Data were expressed as categorical and continuous variables. For categorical data, we used frequencies and percentages. Continuous variables are described as means ± standard deviations (SDs). We used chi-square or Fisher's exact tests as appropriate. Descriptive statistics, cross-tabs, and binary logistic regressions were utilized. Descriptive statistics such as frequency, percentage, and mean were computed. And it was used to analyze participant characteristics. The association was tested using the odds ratio and *p*-value. The final association was declared using the adjusted odds ratio and *p* < 0.05. Descriptive results are presented as proportions (%) with 95% confidence intervals (CIs).

### Ethics statement

This retrospective study was approved by the Biomedical Ethics Review Committee of WCHSU (Ethics Approval No. 2023-1173).

## Results

### Patient demographics and characteristics of the IHOPSs

The baseline characteristics of patients are shown in Table 1. During the study, a total of 351,884 patients received medications. There were no significant sex-based differences among them. The largest group of patients was aged between 36 and 59 years (*n* = 172,271; 48.96%).

**Table 1:** Baseline characteristics of patients (*n* = 351,884).

**Characteristic**	**Value**
**Sex**	**No (%)**
Male	152,937 (43.46%)
Female	198,947 (56.54%)
**Age (years)**	**No (%)**
0–17	6,348 (1.80%)
18–35	95,548 (27.15%)
36–59	172,271 (48.96%)
60–74	60,102 (17.08%)
≥75	17,615 (5.01%)

**Figure 2:**
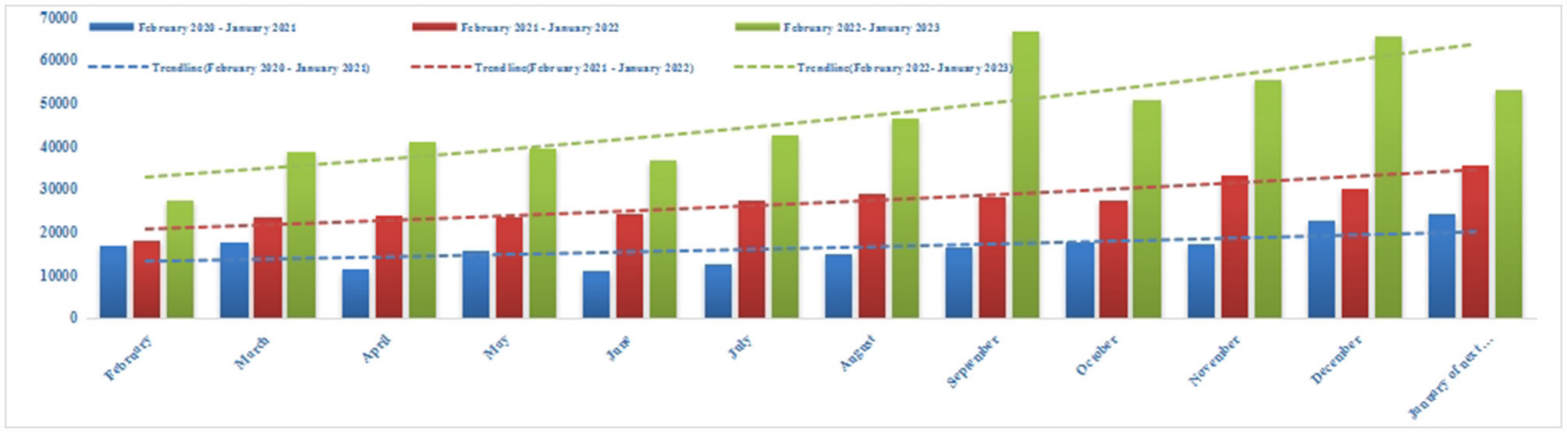
Monthly number of IHOPS medication prescription review items from February 2020 to January 2023.

**Figure 3:**
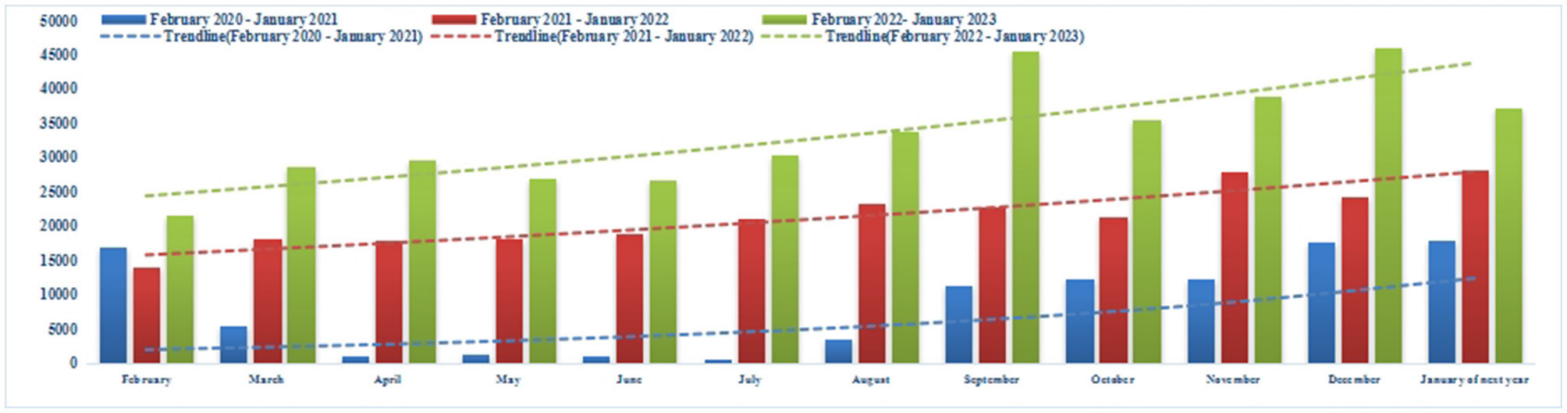
Monthly number of IHOPS medication prescription items from February 2020 to January 2023.

A total of 373,936 Internet hospital prescriptions were delivered between February 2020 and January 2023. Monthly numbers of IHOPS medication prescription review items and delivery prescription items in 2023 were 47,158 and 33,520, respectively (Figures 2, 3). From 2020 to 2023, the number of Internet hospital prescriptions increased by 74.37%. Use of the IHOPS also increased over time, indicating increased acceptance thereof. Even after the COVID-19 pandemic was declared over (December 2022–January 2023), the number of prescriptions increased compared with the same period in each year.

**Figure 4:**
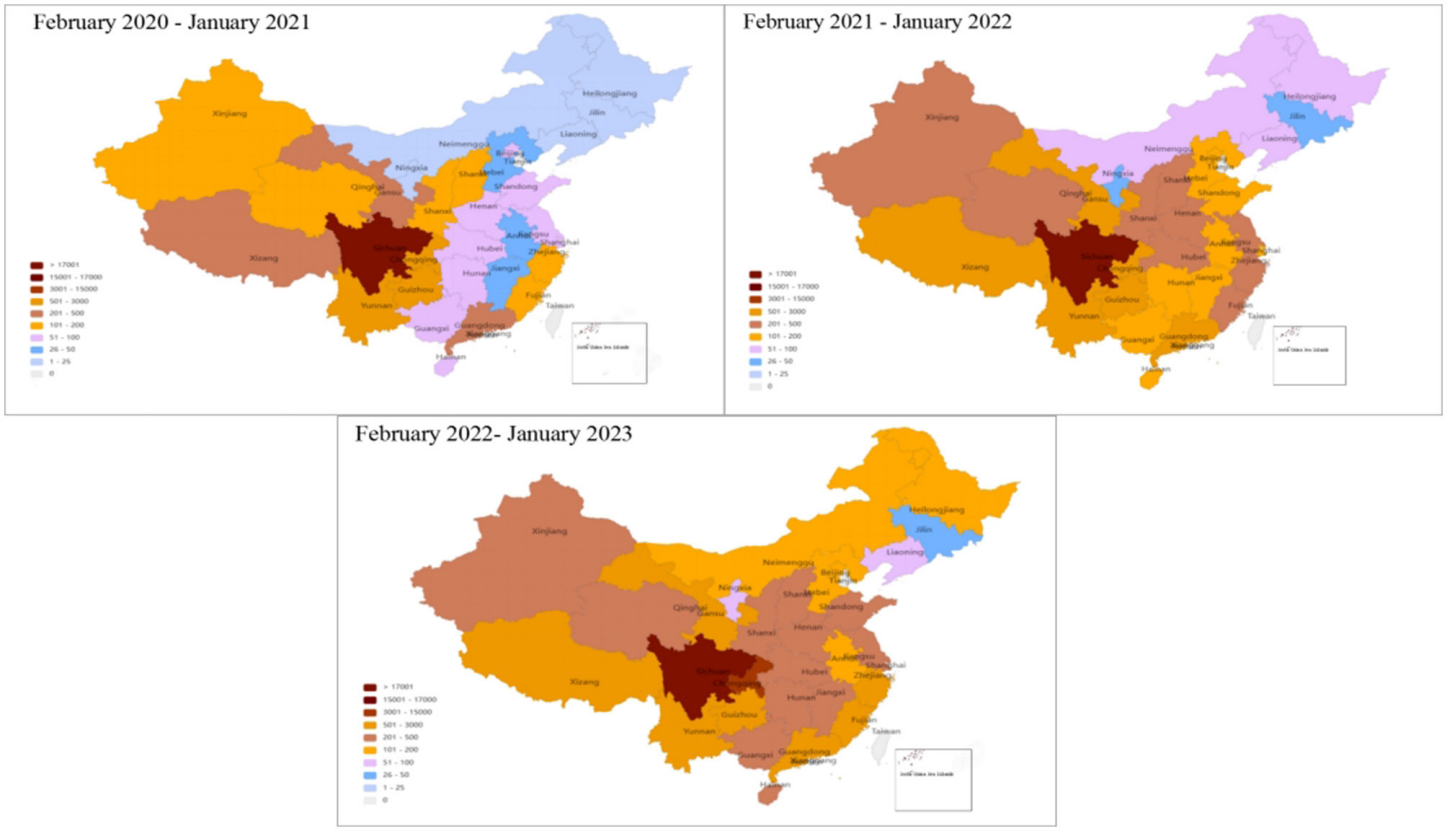
Regional distribution of IHOPS prescription deliveries from February 2020 to January 2023.

The 373,936 prescriptions were delivered to 22 provinces, 5 autonomous regions, and 4 municipalities of China; of these, 4,702 prescriptions required storage at 2–8°C. A total of 90.15% (337,104/373,936) of Internet hospital prescriptions were sent to Sichuan. The top 5 provinces for out-of-province prescription deliveries were Chongqing (*n* = 5,974; 1.6%), Guizhou (*n* = 5,509; 1.47%), Yunnan (*n* = 5,163; 1.38%), Gansu (*n* = 3,227; 0.86%), and Guangdong (*n* = 2,637; 0.71%; Figure 4).

**Figure 5:**
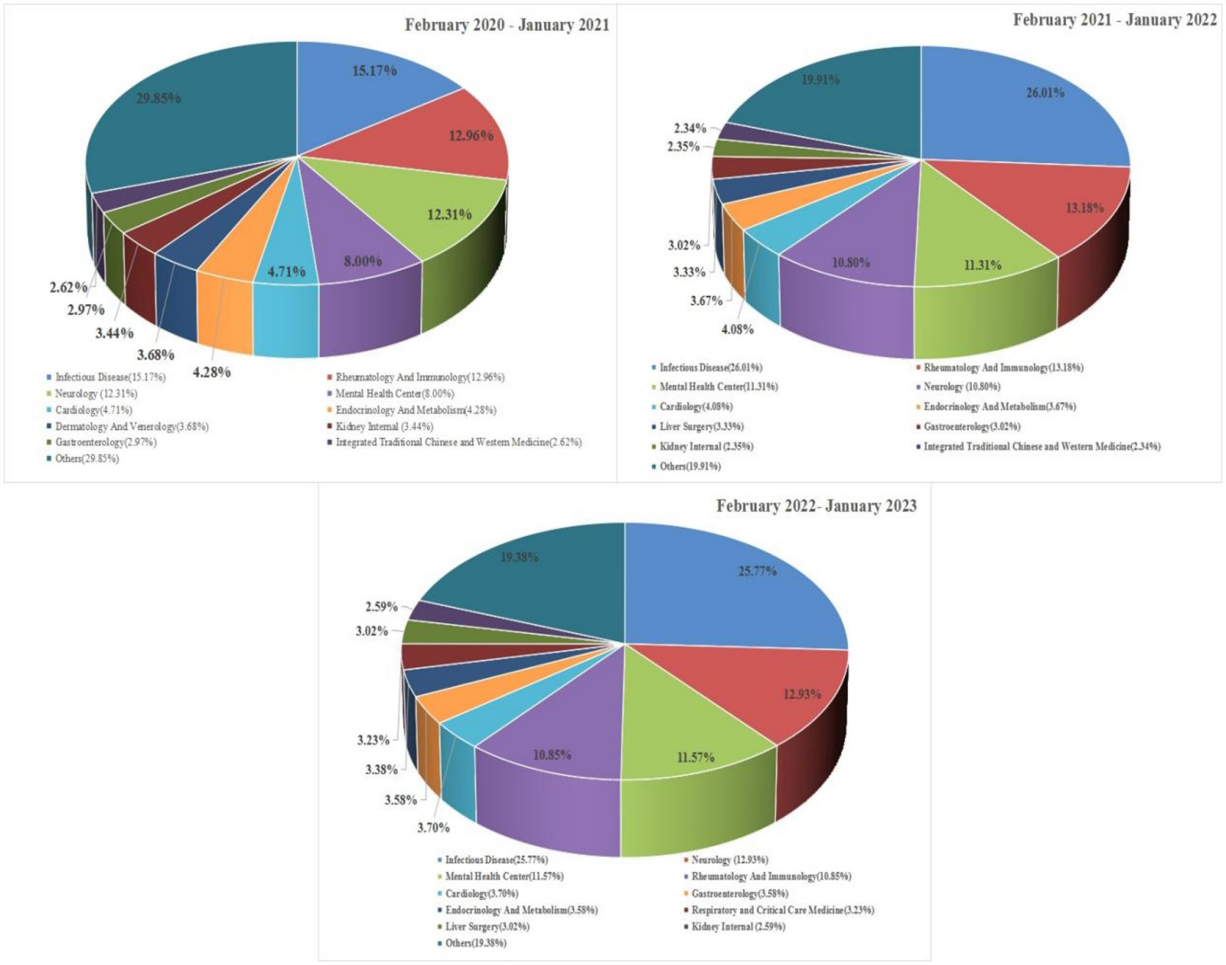
Distribution of the top 10 originating departments for IHOPS prescriptions from February 2020 to January 2023.

The Internet hospital has 75 departments. Figure 5 shows that the departments prescribing the most medications were the infectious diseases department (*n* = 63,903; 17.09%), neurology department (*n* = 45,324; 12.12%), and rheumatology and immunology department (*n* = 4,865; 11.95%). The infectious diseases department ranked first for the 3^rd^ year in a row. The rheumatology and immunology department ranked second in February 2021–January 2022 and February 2022–January 2023, and the neurology department rose to the second place in February 2022–January 2023.

A total of 418,422 diagnoses were represented in our dataset. The top 10 diseases from February 2020 to January 2023 are shown in Table 2. Viral hepatitis B ranked first in February 2021–January 2022 and in February 2022–January 2023, chronic viral hepatitis B ranked second for all three consecutive years, and primary hypertension ranked third in February 2021–January 2022 and in February 2022–January 2023.

**Table 2:** Top 10 diseases from February 2020 to January 2023.

**Time**	**February 2020–January 2021**		**February 2021–January 2022**		**February 2022–January 2023**	
**Category**	**Diagnosis**	**Value (*****n** =* **,148) (*****n*** **%)**	**Diagnosis**	**Value (*****n** =* **61,038) (*****n*** **%)**	**Diagnosis**	**Value (*****n** =* **307,236) (*****n*** **%)**
No.1	Primary hypertension	4,868 (9.71)	Viral hepatitis B	6,785 (11.12)	Viral hepatitis B	22,269 (7.25)
No.2	Chronic viral hepatitis B	3,882 (7.74)	Chronic viral hepatitis B	5,653 (9.26)	Chronic viral hepatitis B	14,033 (4.57)
No.3	Coronary atherosclerotic cardiopathy	2,828 (5.64)	Primary hypertension	5,032 (8.24)	Primary hypertension	10,327 (3.36)
No.4	Rheumatoid arthritis	1,895 (3.78)	Allogeneic kidney transplantation status	2,754 (4.51)	Depressive state	7,313 (2.38)
No.5	Systemic Lupus Erythematosus	1,596 (3.18)	Allogeneic kidney transplantation status	2,480 (4.06)	Coronary atherosclerotic cardiopathy	5,630 (1.83)
No.6	Diabetes	1,383 (2.76)	Coronary atherosclerotic cardiopathy	2,391 (3.92)	rheumatoid arthritis	5,222 (1.70)
No.7	Chronic nephrosis	1,309 (2.61)	Epilepsy	1,650 (2.70)	Allogeneic kidney transplantation status	4,982 (1.62)
No.8	Anxiety state	1,281 (2.55)	Parkinson's disease	1,447 (2.37%)	Parkinson's disease	4,509 (1.47)
No.9	Parkinson's disease	949 (1.89)	Depressive state	1,434 (2.35)	Osteoporosis	3,713 (1.21)
No.10	Diabetic nephropathy	855 (1.70)	Mammary gland malignant tumor	1,114 (1.83)	Systemic Lupus Erythematosus	3,400 (1.11)

The 373,936 delivered prescriptions included 945,172 unique medications. The top 10 delivered prescriptions from February 2020 to January 2023 are listed in Table 3. Entecavir ranked first for all three consecutive years.

**Table 3:** Top 10 delivered medications from February 2020 to January 2023.

**Time**	**February 2020–January 2021**			**February 2021–January 2022**			**February 2022–January 2023**		
**Category**	**Medicine trade name**	**Pharmaceutical dosage form**	**No (%)**	**Medicine trade name**	**Pharmaceutical dosage form**	**No (%)**	**Medicine trade name**	**Pharmaceutical dosage form**	**No (%)**
No.1	Entecavir	Dispersible Tablet	5,414 (3.74%)	Entecavir	Dispersible Tablet	22,589 (8.18%)	Entecavir	Dispersible Tablet	30,707 (5.86%)
No.2	Plaquenil	Tablet	4,483 (3.10%)	Tenofovir Disoproxil Fumarate	Tablet	16,776 (6.08%)	Tenofovir Alafenamide Fumarate	Tablet	18,245 (3.48%)
No.3	Tenofovir Disoproxil Fumarate	Tablet	4,214 (2.91%)	Plaquenil	Tablet	7,214 (2.61%)	Tenofovir Disoproxil Fumarate	Tablet	14,387 (2.74%)
No.4	Calcium carbonate and Vitamin D3	Tablet	4,186 (2.89%)	Calcium carbonate and Vitamin D3	Tablet	6,864 (2.49%)	Plaquenil	Tablet	12,607 (2.40%)
No.5	Alfacalcitol	Tablet	3,859 (2.66%)	Alfacalcitol	Tablet	5,865 (2.12%)	Calcium carbonate and Vitamin D3	Tablet	12,023 (2.29%)
No.6	Methylprednisolone	Tablet	2,976 (2.05%)	Calcitriol	Soft Capsule	5,739 (2.08%)	Alfacalcitol	Soft Capsule	10,546 (2.01%)
No.7	Calcitriol	Soft Capsule	2,327 (1.61%)	Methylprednisolone	Tablet	4,400 (1.59%)	Prednisone Acetate	Tablet	10,408 (1.99%)
No.8	Atorvastatin Calcium	Tablet	2,244 (1.55%)	Sertraline Hydrochloride	Tablet	3,799 (1.38%)	Atorvastatin Calcium	Tablet	7,473 (1.43%)
No.9	Aspirin	Enteric-coated Tablet	2,170 (1.50%)	Compound Biejia Ruangan	Tablet	3,760 (1.36%)	Compound Biejia Ruangan	Tablet	7,114 (1.36%)
No.10	Leflunomide	Tablet	2,069 (1.43%)	Prednisone Acetate	Tablet	3,663 (1.33%)	Sertraline Hydrochloride	Tablet	6,523 (1.24%)

The top 10 medications accounted for 25.88% (244,644/945,172) of total medications. The average prescription cost was RMB ¥189.20 (range, RMB ¥0.17–21,825.00). Most medications were used to treat infectious diseases, diseases of the immune system, cardiovascular diseases, and neurological diseases, which was consistent with the departmental and diagnostic distributions of IHOPS prescriptions.

### Medication quality and safety analysis for the IHOPSs

Pharmacists reviewed a total of 552,121 prescriptions representing 1,094,039 medications using the rational medication monitoring system; 470,969 prescriptions were approved, while 81,152 prescriptions were not approved during review. Of all reviewed prescriptions, 92.58% (81,152/1,094,039) were deemed appropriate. If a prescription was not approved, the pharmacist would communicate electronically with the doctor, who would confirm or re-prescribe the medication. Pharmacists double-checked all the medications when dispensing. The checks revealed the existence of prescription review errors from February 2020 to January 2023 (Table 4). There were a total of six errors, yielding an error rate of 0.0011% for prescription review.

**Table 4:** Analysis of prescription review errors.

**Time**	**Medicine**	**Error types**	**Unreasonable types**	**Concrete issue**
July 29, 2020	Kangfuxin Solution	Omission intervention	Inappropriate dosage	The usual oral dosage of Kangfuxin liquid is 10 ml, which is staggered into appropriate amount.
August 30, 2020	Hydrochlorothiazide Tablets	Omission intervention	Inappropriate dosage	Dosage staggered to 0.01 tablets.
February 14, 2021	Budesonide and formoterol Fumarate Powder for Inhalation (Symbicort Turbuhaler)	Omission intervention	Diagnosis writing errors	The diagnosis misstates COPD as brain obstruction.
March 27, 2021	Bromocriptine Mesilate tablets	Misintervention	/	Pharmacists intervene in the manual indication of Parkinson's disease.
May 13, 2021	Tenofovir Disoproxil Fumarate Tablets	Omission intervention	Inappropriate dosage	Normal dosage 300 mg staggered to 0.5 mg.
September 21, 2022	Acarbose Capsules	Omission intervention	Repeated medication	They have the same mechanism of action.
	Miglitol Tablets			

For the 373,936 delivered prescriptions, the number of dispensing errors was 3, and the dispensing error rate was 0.0008%. Specific analysis of dispensing errors is shown in Table 5. All dispensing errors were found during daily inventory and promptly corrected via contact with the patients.

**Table 5:** Analysis of dispensing errors.

**Time**	**Error types**	**Error medicine**	**Concrete issue**	**Reason**
May 30, 2022	Wrong medicine, medication looks similar	Olanzapine Tablets	The specification of olanzapine is 5 mg, and it is mistakenly taken as 10 mg.	Habitual processing
December 15, 2022	Omission of medicine	Leucogen Tablets	There were omissions in medicine dispensing.	Poor concentration
May 9, 2023	Wrong patient	/	The pharmacist pasted the drug delivery waybill incorrectly, resulting in the wrong delivery of patients.	Inadequate screening of patient

### Economic-value analysis of the IHOPSs

According to Yang et al. (8), the economic value is calculated as follows. The average annual wages of employed persons living in private urban housing units nationwide in 2020, 2021, and 2022 were RMB ¥57,727 (¥158/day) (9), ¥62,884 (¥172/day) (10), and ¥65,237 (¥179/day), respectively (11). A second-class high-speed rail seat ticket costs RMB ¥0.31/km (12). Based on the median distance from the patient delivery address to the hospital, medications usually take 0.5 days to travel 50 km, 1 day to travel 100–500 km, 1.5 days to travel 500–750 km, 2 days to travel 750–1,500 km, and 3 days to travel 1,500–3,000 km. The IHOPSs saved patients >320,377 days (7,689,048 h) and RMB ¥94.05 million from February 2020 to January 2023, as shown in Table 6.

**Table 6:** Economic-value analysis of the IHOPS from February 2020 to January 2023.

**Time**	**February 2020–January 2021**	**February 2021–January 2022**	**February 2022–January 2023**
The round-trip time (day)	48,096	108,692	163,589
The Wage loss of round trip (RMB)	7,599,168	18,695,024	29,282,342
The round-trip transportation cost (RMB)	5,388,410	13,503,210	19,581,219
Total cost (RMB)	12,987,578	32,198,234	48,863,561

### Patient satisfaction with the IHOPSs

We conducted a simple 5-point random-sampling survey to investigate the satisfaction of 322 patients who used the IHOPSs. In total, 95.96% (309/322) of these patients provided scores of 5, and another 4.04% (13/322) provided scores of 4. The patients were also very willing to recommend the IHOPSs to other patients (100%).

## Discussion

Increasing digitization and Internet technology have changed the methods people use to seek medical services (13), with an increase in the use of technology, often to reduce face-to-face contact (14, 15). The unbalanced allocation of medical resources and the outbreak of COVID-19 (16) promoted the growth of and improvements to more-convenient Internet medical practices (17). An increasing number of scholars have studied Internet pharmacies. Chen et al. and Ding et al. assessed the characteristics, initial impact, and acceptance of cloud pharmacies during the COVID-19 outbreak in economically developed South China (18, 19). Telepharmacy services and expertise are accessible in a number of other nations as well. Hammour et al. (20) investigated the Internet pharmacy drug delivery platform in Jordan and found it to be efficient and convenient in delivering medications to patients on time during the COVID-19 outbreak. Several advantages of these new services have been reported. They save time and money, especially for older adult, who more frequently visit healthcare institutions. They also provide broad coverage of pharmaceutical care services, particularly in economically challenged areas, and help decrease healthcare inequities (21). The above-cited studies focused on the benefits of Internet hospital pharmacies, but safety and security were not included in those benefits; nor was there a focus on the characteristics of Internet hospital pharmacies after the COVID-19 pandemic was controlled and declared over.

The current study ran for 3 years, and the research data included 373,936 prescriptions. The IHOPS of WCHSU has been officially online since February 2020. From opening to January 2023, it was used by >350,000 patients, with an average of 365 patients per day. The number of prescriptions has increased year over year rather than decreasing significantly after the COVID-19 pandemic, indicating that this model is suitable and convenient for patients purchasing drugs on an everyday basis (Figures 2, 3).

From the departments issuing the most prescriptions (Figure 5), the diseases most commonly treated by IHOPS medications (Table 2), and delivered medications (Table 3), it can be seen that patients with chronic diseases requiring long-term medication are the main customers of the IHOPS; its services could also help patients with chronic diseases easily renew their prescriptions and receive their medication.

The most important advantages of Internet healthcare are time and cost savings for patients. In this study, we calculated that the IHOPS saved patients more than 320,377 days (7,689,048 h) and RMB ¥94.05 million over the 3 years of the study (Table 6). The random questionnaire survey of IHOPS patients suggested that the majority had high acceptance of IHOPS.

In general, because the IHOPS is online, it improves access to quality pharmacy resources without geographical limitations. This was especially true during the COVID-19 pandemic, which played an enormous role in popularizing such platforms. Even in the current post-pandemic era, IHOPSs have the potential to address persistent obstacles to primary pharmaceutical service, including scarcity of trained pharmacists, the difficulties of patient transportation, and personnel costs. Widespread use of the IHOPS could help alleviate pressure on offline pharmacists, giving them the time and resources to provide other professional services. Development of Internet hospitals could become a megatrend for other offline hospitals. The long 3-year period and extensive research data of this study allowed us to analyze the characteristics, medication safety and security, economic value, and acceptance of the IHOPS in depth. Our results can serve as a useful reference for other hospitals to support the development of Internet hospital pharmaceutical care.

Based on our findings, there are some suggestions that deserve further discussion to provide policy enlightenment for the development of the IHOPS. First, the main work of the IHOPS is not only to deliver drugs, but pharmacists should use the mode to provide patients with online pharmaceutical care such as online medication education, optimal medication design, and chronic disease management. Second, strengthen the professional management of pharmaceutical logistics personnel. Medical logistics is different from ordinary logistics. Logistics and distribution need to be guided and managed by personnel with a pharmaceutical background. Third, to establish and improve the quality standards of pharmacy service in internet hospital pharmaceutical care. There is an urgent need to standardize the implementation standards to ensure that patients can access safe and effective Internet + outpatient pharmacy services.

Unlike in previous studies, we have researched that how to ensure the safety and security of IHOPS medications, an effort that achieved remarkable results. The error rates of prescription review and dispensing were 0.0011% (Table 4) and 0.0008% (Table 5), respectively. Meanwhile, no complaints or disputes caused by medication quality or errors arose. These results showed that our control measures played a role in the safety and security of IHOPS. In addition, the model is simple and easy to popularize and can be used for reference by other hospitals.

Some limitations to IHOPSs remain to be improved, such as the lack of face-to-face interaction between healthcare practitioners (for example, pharmacists) and patients and higher risks to data confidentiality and integrity. In our calculations of economic value, we did not include the costs of accommodation and food, meaning that we might have underestimated the economic value of the IHOPS. In addition, using high-speed rail fares to calculate transportation tolls limited the scope of its potential economic value across the globe. We also need to compare the advantages and disadvantages with patients who do not use the IHOPS mode. Because the IHOPS is a new model of pharmaceutical service, more research is needed to verify its effectiveness and quality.

This new pharmaceutical service has brought numerous benefits to patients. In the near future, a new pharmacy service based on artificial intelligence (AI), encompassing such functions as medication reminders and medication tracking, will be developed. Clear, simple instructions are necessary to improve the acceptance of IHOPSs by older people. Meanwhile, under the national health department support, a regional internet prescription flow platform based on the mode of IHOPS and can share internet prescription information. Finally, standardizing the operational process and management of IHOPSs will require official regulations.

## Conclusions

The Department of Pharmacy at WCHSU has creatively expanded its capabilities by identifying ways to improve the use of health technology and management measures. This will ensure safe medication delivery while improving access to quality pharmacy resources. Our findings suggested that the IHOPS could provide efficient, cost-saving, and convenient pharmaceutical services to patients with chronic diseases, bypassing the limitations of geography and time. This study on the characteristics of pharmaceutical services and the establishment of a quality and safety model can serve as a useful reference for policymakers to support the development of Internet pharmaceutical services.

## Data Availability

The original contributions presented in the study are included in the article/supplementary material, further inquiries can be directed to the corresponding authors.
